# Case Report: Toxic tubulointerstitial nephropathy with lipofuscin deposition – the potential cause of occupational Bisphenol-A exposition

**DOI:** 10.3389/pore.2025.1612046

**Published:** 2025-07-07

**Authors:** László Bidiga, Tamás Csonka, Gábor Méhes, Csilla Markóth, Dávid Hutkai, János Mátyus

**Affiliations:** ^1^ Department of Pathology, Faculty of Medicine, University of Debrecen, Debrecen, Hungary; ^2^ Division of Nephrology, Department of Internal Medicine, Faculty of Medicine, University of Debrecen, Debrecen, Hungary

**Keywords:** bisphenol A (BPA) exposure, nephrotoxicity, chronic kidney disease, lipofuscin accumulation, workplace safety

## Abstract

This case study delves into the link, between exposure to Bisphenol A (BPA) and kidney issues filling a gap in human focused research found in studies. The individual, a 72-year man with a history of BPA exposure in a plastics manufacturing facility experienced a gradual decline in kidney function over 18 months. Medical tests showed kidney disease with a buildup of lipofuscin in renal tubular cells upon examination. This discovery suggests a connection between BPA exposure and kidney damage underscoring the need for investigation. The lack of human based evidence highlights the importance of research to understand the toxic effects of BPA on the kidneys. In addition, to its implications this case emphasizes the importance of improving safety protocols and raising awareness among healthcare professionals in relevant work environments to reduce potential health risks associated with BPA exposure.

## Introduction

Bisphenol A (BPA) and its analogues are widely used in consumer products such as plastics, food packaging, thermal paper receipts, and dental sealants. These compounds can penetrate into food, water, and the environment, leading to ongoing human exposure [1, 2]. Increasing evidence links BPA exposure to adverse health effects, including potential kidney-related outcomes, although the precise mechanisms remain unclear.

Epidemiological studies have reported associations between BPA levels in blood or urine and an increased risk of kidney dysfunction [3]. In response to growing concerns, the European Food Safety Authority (EFSA) has set a tolerable daily intake (TDI) of 4 μg/kg body weight/day [4]. Despite regulatory efforts, chronic exposure persists and may lead to renal injury through mechanisms such as oxidative stress, inflammation, and fibrosis [5].

The kidneys, essential for waste filtration and fluid homeostasis, are particularly susceptible to damage from toxicants like BPA. BPA has been shown to act as an endocrine disruptor, mimicking estrogen and interfering with hormone-regulated functions, which may contribute to hypertension, diabetes, and other risk factors for chronic kidney disease. Animal studies suggest that BPA exposure induces structural and functional changes in the kidneys, including tubular injury and fibrotic remodeling [5].

Moreover, BPA may contribute to the accumulation of lipofuscin, a pigment composed of oxidized proteins and lipids often associated with cellular aging and oxidative damage. Lipofuscin accumulates primarily in long-lived, postmitotic cells such as neurons, myocytes and renal tubular epithelial cells, impairing lysosomal and mitochondrial function and potentially exacerbating age-related renal decline [6]. Although the role of BPA in lipofuscin accumulation has been explored in animal models, human data on this specific nephrotoxic pathway are lacking.

This case report attempts to address this gap by presenting evidence of lipofuscin accumulation in human renal tubular epithelial cells in association with documented BPA exposure. The findings contribute to understanding how environmental toxicants like BPA may influence renal aging and pathology at the cellular level.

## Case presentation

In October 2023, a 72-year-old man presented to our nephrology clinic with a progressive decline in kidney function, most recently evidenced by an estimated glomerular filtration rate (eGFR) of 28 mL/min/1.73 m^2^. The patient’s family history was notable for pancreatic cancer in his mother and cardiovascular disease in his father (hypertension, diabetes, and myocardial infarction), but there was no known familial kidney disease. He was a non-smoker with only minimal past tobacco use, and consumed alcohol occasionally. His hypertension and type 2 diabetes mellitus, diagnosed a decade earlier, were both well-controlled. He was on long-term medication for his hyperlipidemia condition along with his hyperuricemia and benign prostatic hyperplasia.

A decreased eGFR of 46 mL/min/1.73 m^2^ had been initially noted in November 2021, prompting suspicion of nephrosclerosis by his general practitioner due to the absence of urinary abnormalities. By April 2023, his kidney function had further declined to 32 mL/min/1.73 m^2^, and ultrasonography revealed mild bilateral renal parenchymal reduction to 13 mm. Laboratory work-up, including urine analysis and serologic testing, showed no significant pathology. An ophthalmological examination ruled out diabetic or hypertensive retinopathy.

The patient had been taking NSAIDs—specifically diclofenac and aceclofenac—intermittently for musculoskeletal pain due to lumbosacral spondyloarthropathy, raising concerns for NSAID-related nephrotoxicity. Following medical advice, he discontinued NSAIDs and transitioned to tramadol and occasional muscle relaxants. Despite this change, eGFR declined further to 28 mL/min/1.73 m^2^ over the following 4 months. A second evaluation from a nephrologist was performed in October 2023. Physical examination revealed obesity, his BMI was 43. Doppler sonography showed preserved renal perfusion and no evidence of renal artery stenosis or obstruction. The patient showed no symptoms of analgesic nephropathy according to his clinical presentation.

Further laboratory evaluation detected a slightly decreased hemoglobin level (132 g/L) and a low IgM level (0.27 g/L), prompting a myeloma workup. Serum protein electrophoresis showed a monoclonal IgG lambda component of 2.6 g/L, however, the serum free kappa/lambda ratio remained within normal range (0.697). Despite the absence of proteinuria (urine protein/creatinine ratio 5 mg/mmol) and hematuria, a kidney biopsy was indicated and done to elucidate the cause of kidney function decline.

The renal biopsy revealed preserved glomerular architecture. However, there was moderate to severe interstitial fibrosis and tubular atrophy (IFTA), with granular cytoplasmic inclusions predominantly in the proximal tubular epithelial cells. These findings raised suspicion for chronic toxic exposure affecting the tubular compartment. Special histochemical stains were performed to identify potential endogenous or exogenous pigments. Prussian blue (iron), rhodamine (copper), and Fouchet (bile) stains were negative. In contrast, Sudan Black, Oil Red O, and Toluidine Blue stains were positive, indicating the presence of lipid-rich material.

**FIGURE 1:**
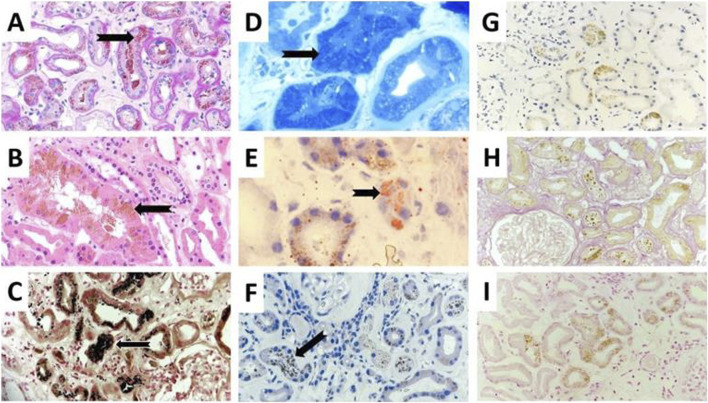
Presents a comparative view with **(A)** showcasing a routine periodic acid-Schiff (PAS) stain at ×200 magnification and **(B)** displaying a hematoxylin and eosin (HE) stain at ×400 magnification with intraepithelial lipofuscin brown pigmentation (black arrows). The special stains like: Masson Fontana **(C)**, Touluidine blue **(D)**, Oil Red-O **(E)** and Sudan black **(F)** for lipid content evaluation displayed positive staining (200x, black arrows). Other possible brownish pigments like copper with Rhodamin stain **(G)**, bile with Fouchet stain **(H)** and iron with Prussian Blue stain **(I)** all yielding negative outcomes (200x).

**FIGURE 2:**
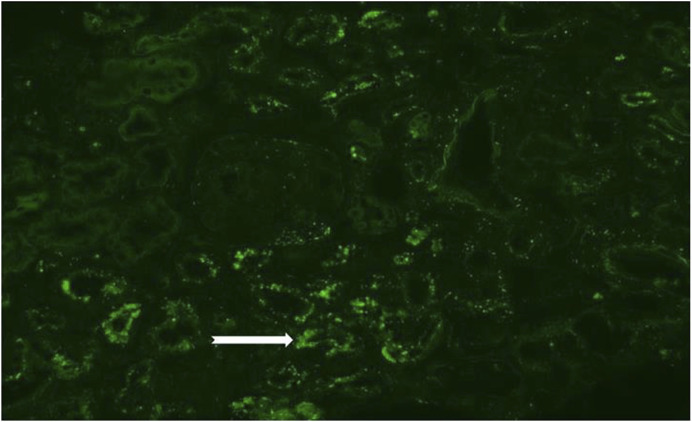
Presents an immunofluorescence examination, showcasing a control slide with noticeable green autofluorescent cytoplasmic granules in the tubular epithelial cells (200x, white arrow).

Masson-Fontana staining confirmed the presence of lipofuscin granules within the tubular epithelial cells (Figure 1). Immunofluorescence studies revealed aspecific positivity for C3c in small vessels and for IgG, kappa, and lambda in tubular resorption granules and casts. No monoclonal immunoglobulin deposition was identified, thus ruling out monoclonal gammopathy of renal significance (MGRS). Control slides revealed green autofluorescent cytoplasmic granules in epithelial cells (Figure 2).

**FIGURE 3:**
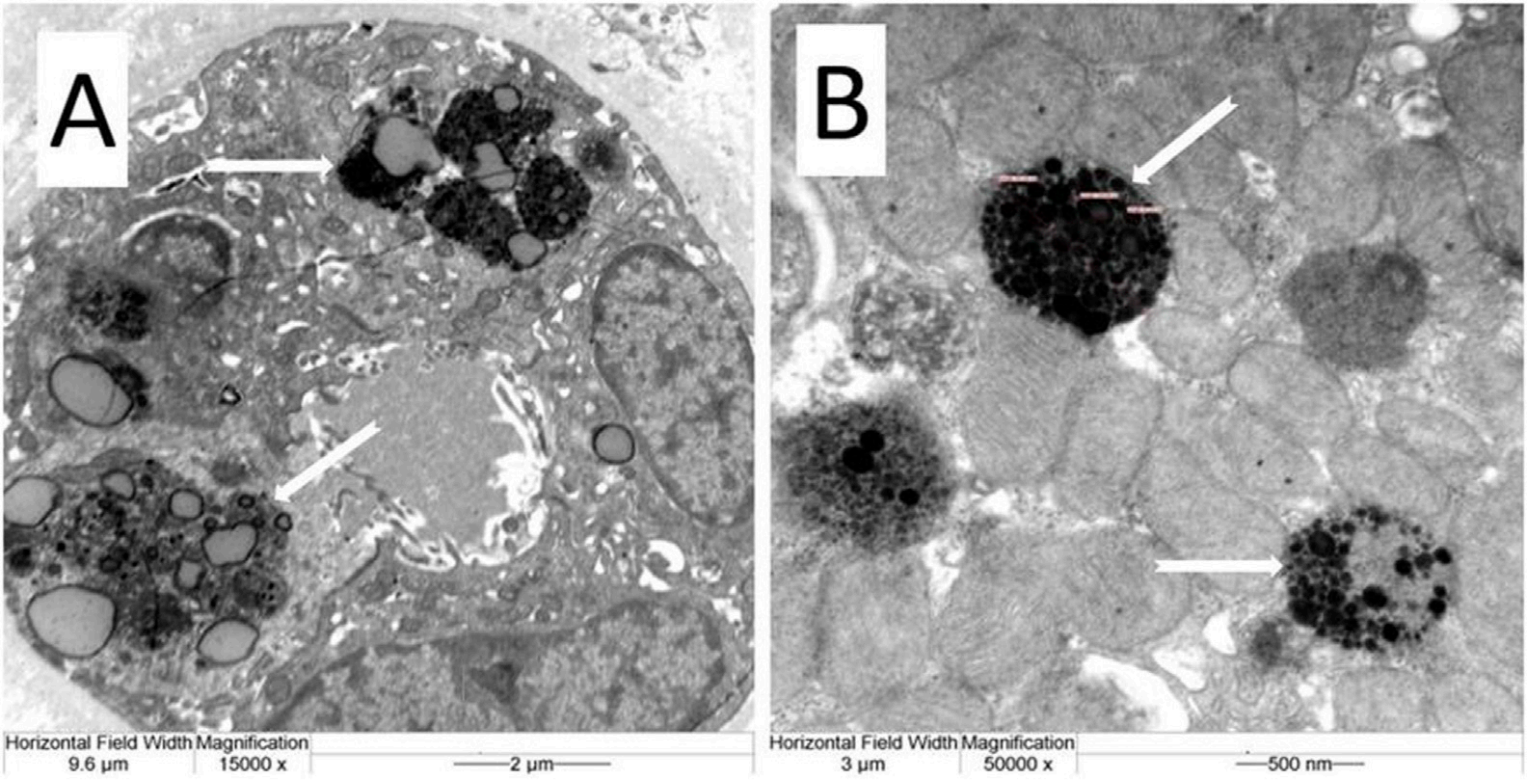
Presents electron microscopic ultrastructural images capturing the tubular epithelial cell lipofuscin granules. At 15,000x magnification **(A)** and further magnified at 50,000x **(B)**, these detailed images provide a closer look at the cellular structures affected by lipofuscin accumulation.

Electron microscopy further demonstrated characteristic electron-dense lipofuscin granules within the proximal tubular epithelial cytoplasm at magnifications of 15,000x and 50,000x (Figure 3).

The exclusion of other potential causes of lipofuscin accumulation—such as aging, vitamin E deficiency, amiodarone, aluminum exposure, phenacetin/paracetamol use, uremia or transplant rejection—alongside the patient’s occupational background in a BPA-associated environment (plastic manufacturing open offices) raised concerns about environmental BPA exposure. While the patient was not directly involved in manufacturing, but workplace proximity allowed chronic low-level exposure to be considered. This, coupled with prior NSAID use potentially exacerbating oxidative stress, likely contributed to lipofuscin buildup.

Our patient was not exposed to other known lipofuscin-inducing agents. Based on clinical suspicion and the unusual histological findings, targeted genetic testing was recommended. Genetic analysis was performed on peripheral blood at the Department of Medical Genetics, University of Debrecen, Debrecen, Hungary. A custom-designed 33-gene next-generation sequencing (NGS) panel was applied, developed in-house by the Department for the detection of common hereditary renal disorders, using the Twist Custom Panel kit (Twist Bioscience) and the Illumina NGS platform. The complete list of the 33 genes included in the renal gene panel is provided in Supplementary Table S1.

As part of management, diclofenac patches and other NSAIDs were permanently discontinued. Pain management was optimized with tramadol. Given the patient’s persistent obesity, worsening renal function, and use of metformin, his dosage was reduced to 500 mg every other day. Oral semaglutide therapy was initiated. At follow-up, kidney function remained stable (eGFR unchanged), and his monoclonal IgG lambda component, blood pressure, HbA1c, LDL-cholesterol, and serum uric acid levels remained within target ranges. His ongoing medications included irbesartan, amlodipine, carvedilol, indapamide, furosemide, potassium chloride, esomeprazole, allopurinol, rosuvastatin, metformin, desloratadine, and vitamin D3.

This complex clinical course highlights a multifactorial decline in kidney function with a unique histopathological signature of lipofuscin accumulation likely related to combined environmental BPA exposure and oxidative stress. While a definitive causative relationship cannot be conclusively proven, the exclusion of other known causes and the novel histological features support the plausibility of this association.

## Discussion

Lipofuscin accumulation in renal tubular epithelial cells is generally considered a histological marker of cellular aging. It reflects cumulative oxidative stress and lysosomal dysfunction and is often seen in elderly individuals as a part of physiological aging. However, its pronounced presence in the proximal tubules of this patient, combined with the absence of similar findings in over 2,000 kidney biopsies performed at our institution in the past 15 years—prompted a broader differential diagnosis.

Lipofuscin can also accumulate in pathological states such as vitamin E deficiency, chronic oxidative stress (as observed in uremia or transplant rejection), and exposure to certain drugs, notably amiodarone. The patient had no history of exposure to such medications and no overt micronutrient deficiencies were documented. Notably, the patient did have chronic conditions that could contribute to tubulointerstitial damage and oxidative stress—including type 2 diabetes, obesity, and a history of NSAID (diclofenac) use. Diclofenac is chemically known to increase reactive oxygen species and induce apoptosis [7], although lipofuscin deposition has not been previously associated with NSAIDs, unlike other analgesics.

While some genes implicated in autophagic dysfunction and lipofuscin accumulation (e.g., *SMP30*, *LRRK2* [8]) were not tested, no alternative genetic predisposition could be established. Each of these factors has been associated with progressive kidney injury and must be considered when interpreting the findings.

The kidney biopsy showed moderate interstitial fibrosis and tubular atrophy (IFTA), but the glomeruli were preserved and there was no significant glomerular pathology. Importantly, there was no evidence of significant arterial or arteriolar sclerosis, suggesting that nephrosclerosis was not the dominant process driving renal decline in this case. Additionally, the interstitial compartment showed no notable inflammatory infiltrate, making tubulointerstitial nephritis or immune-mediated processes unlikely contributors.

Given this context, the presence of dense, autofluorescent, lipofuscin-positive granules—confirmed by Masson-Fontana and lipid stains—in proximal tubular epithelial cells becomes particularly noteable. Electron microscopy confirmed the finding, revealing well-demarcated electron-dense granules consistent with lipofuscin morphology.

While a definitive causal relationship between BPA exposure and renal injury in this patient cannot be established, prior animal studies have shown that BPA can induce oxidative stress, interfere with lysosomal degradation, and lead to lipofuscin accumulation in renal tubular epithelial cells [9–12]. BPA has also been implicated in disrupting mitochondrial function, inducing ferroptosis, and changing autophagic flux in kidney tissue [5, 7, 13, 14]. These mechanisms offer biological plausibility that supports a potential link between environmental BPA exposure and the unusual histologic features observed in this patient.

While the patient had plausible environmental risk factors—use of plastic containers, consumption of packaged food, and handling of thermal paper receipts—no BPA levels were assessed in blood or urine. Similarly, no systemic symptoms or biochemical alterations commonly associated with BPA exposure (e.g., hormonal disruption, elevated blood pressure beyond his baseline, or features of metabolic syndrome) were reported, beyond his known comorbidities.

This case therefore illustrates a possible, but not definitive, association between lipofuscin accumulation and BPA exposure. It underscores the importance of considering environmental toxins in the differential diagnosis of atypical renal histological findings, especially in the absence of classic causes such as drug toxicity or severe diabetes-related injury. Further studies are required to elucidate the frequency and clinical relevance of lipofuscin deposition in aging kidneys and to confirm whether BPA or similar environmental agents can induce such changes in humans.

## Conclusion

In summary, this case of a 72-year-old man with progressive chronic kidney disease and biopsy-proven lipofuscin accumulation in proximal tubular epithelial cells raises the possibility of a link between prolonged BPA exposure and renal injury. The findings suggest that BPA-induced oxidative stress and autophagy impairment may contribute to tubular damage and cellular aging, as reflected by lipofuscin deposition.

Lipofuscin may serve as a useful histologic marker of environmental toxin exposure and early tubular injury. Given the widespread presence of BPA in consumer products, further investigation into its nephrotoxic potential is urgently needed. Regulatory strategies aimed at reducing BPA exposure may play a preventive role in diminishing the risk of chronic kidney disease, particularly in vulnerable populations. Continued research into environmental nephrotoxins and their interaction with cellular aging pathways is essential to protect kidney health in the broader population.

## Data Availability

The raw data supporting the conclusions of this article will be made available by the authors, without undue reservation.
