# Supplementary material for: Case Report: Toxic tubulointerstitial nephropathy with lipofuscin deposition – the potential cause of occupational Bisphenol-A exposition
Source: Pathol Oncol Res. 2025 Jul 7;31:1612046. doi: 10.3389/pore.2025.1612046 (PMC12277192; doi:10.3389/pore.2025.1612046)
Supplement: Supplementary file 1 [file Table1.docx]

# Supplementary Table 1 – Genes Included in the Nephropathy Gene Panel

| Gene Symbol | Full Gene Name (where applicable) |
| --- | --- |
| ACTN4 | Actinin Alpha 4 |
| ATP6V0A4 | ATPase H+ Transporting V0 Subunit A4 |
| ATP6V1B1 | ATPase H+ Transporting V1 Subunit B1 |
| CA2 | Carbonic Anhydrase 2 |
| CLCN5 | Chloride Voltage-Gated Channel 5 |
| COL4A3 | Collagen Type IV Alpha 3 Chain |
| COL4A4 | Collagen Type IV Alpha 4 Chain |
| COL4A5 | Collagen Type IV Alpha 5 Chain |
| COQ8B | Coenzyme Q8B |
| DNAJB11 | DnaJ Heat Shock Protein Family Member B11 |
| EYA1 | EYA Transcriptional Coactivator and Phosphatase 1 |
| FGF23 | Fibroblast Growth Factor 23 |
| FN1 | Fibronectin 1 (rs137854488 variant) |
| FOXI1 | Forkhead Box I1 |
| HNF1B | Hepatocyte Nuclear Factor 1 Beta |
| INF2 | Inverted Formin 2 |
| LMX1B | LIM Homeobox Transcription Factor 1 Beta |
| NPHS1 | Nephrin |
| NPHS2 | Podocin |
| OCRL | Inositol Polyphosphate-5-Phosphatase |
| PAX2 | Paired Box 2 |
| PHEX | Phosphate Regulating Endopeptidase Homolog X-Linked |
| PKHD1 | Polycystic Kidney and Hepatic Disease 1 |
| REN | Renin |
| SEC61A1 | Sec61 Translocon Alpha 1 Subunit |
| SLC12A3 | Solute Carrier Family 12 Member 3 |
| SLC22A12 | Solute Carrier Family 22 Member 12 |
| SLC4A1 | Solute Carrier Family 4 Member 1 |
| SLC4A4 | Solute Carrier Family 4 Member 4 |
| TRPC6 | Transient Receptor Potential Cation Channel Subfamily C Member 6 |
| UMOD | Uromodulin |
| WDR72 | WD Repeat Domain 72 |
| WT1 | Wilms Tumor 1 |
